# Sesame effects on testicular damage in streptozotocin-induced diabetes rats

**Published:** 2013

**Authors:** Fereshteh Khaneshi, Ozra Nasrolahi, Shahriar Azizi, Vahid Nejati

**Affiliations:** 1 ***Department of Biology, Faculty of science, Urmia University, Urmia, I. R. Iran***; 2 ***Department of Anatomy, School of Medicine, Mashhad University,Mashhad,I. R. Iran***; 3 *Department of Comparative Histology and Embryology,Faculty of Veterinary Medicine, Urmia University, Urmia, I. R. Iran*

**Keywords:** Diabetes, Sesame, Spermatogenesis, Testis

## Abstract

**Objective(s): **Reproductive dysfunction is a consequence of diabetes. Diabetes is associated with changes in testicular tissue. Sesame oil contains large amounts of polyunsaturated fatty acids and lignin with antioxidant activity, vitamin E, and monounsaturated fatty acid (MUFA). The present study investigated the effects of sesame on testis histology and male reproductive parameters in streptozotocin-induced diabetic rats.

**Materials and Methods: **Thirty mature male Wistar rats were randomly divided into three groups, i.e., control (C), diabetic-control (DC), and sesame-treated diabetic rats (SD). Diabetes was induced by a single dose of streptozotocin (65 mg/kg; i.p). The animals were treated by a single intraperitoneal sesame extract injection (100 mg/kg b.w.) once daily for 6 weeks.

**Results: **The biochemical analysis revealed that the diabetes resulted in significant (p<0.05) reduction in spermiogenesis, testosterone, LH, and FSH levels. Light microscopic analysis showed remarkable (p<0.05) reduction in STD (seminiferous tubules diameter), SPI (spermatogenesis index) thickness of the epithelium, and significant increase in thickness of the interstitial tissue in the diabetic group compared with the control group. Simultaneous administration of the sesame could fairly up-regulate testosterone, LH, and FSH of the animals in this group. However, some differences were manifested with improved histological features as thickness of the epithelium, seminiferous tubules diameter, and spermatogenesis index.

**Conclusion: **These data demonstrated that sesame significantly improved diabetes complication in rat testis. This study suggested that sesame might have a protective effect against oxidative stress-induced impaired testicular functions in diabetic rats.

## Introduction

Diabetes has been associated with reproductive impairment of both men and women. About 90% of diabetic patients have turbulence in sexual function that includes decrease in libido, impotence, and infertility (Shi-Liang et al., 2001 ▶; Jiang et al., 1996 ▶). Previous studies showed that two factors, increased oxidative stress and changes in antioxidant capacity are playing an important role in the pathogenesis of chronic diabetes mellitus (Baynes et al., 1999 ▶; Wolff et al., 1991). Streptozotocin (STZ) is a cytotoxic substance that induces diabetes mellitus in experimental animals. Moreover, it causes testicular dysfunction and degeneration in animal models (Shrilatha et al., 2007 ▶).

Sesame (*Sesamum indicum* L.) oil contains large amounts of polyunsaturated fatty acids that are useful for the body. On the other hand, it contains lignin with antioxidant activity, vitamin E, and monounsaturated fatty acid (MUFA). It can also be resistant to lipid oxidation as an antioxidant to remove hydroxyl, proxy radicals, and thus acts to control lipid peroxidation (Sankar et al., 2006 ▶; Kang et al., 2000 ▶). Lipid peroxidation is a reaction that is induced by oxidative stress. This reaction in particular occurs in tissues with rich membranes of activates fatty acids. 

Sesame seed is considered as a healthy diet and is traditionally used in the Middle East and Japan (Kang et al., 1998 ▶). Sesame seed is rich in oil (about 50%) and protein (about 20%) and diverse lignin as sesamin and sesaminol (main lignin), about 1.5% (Rong et al., 2005 ▶). The predominant fatty acids in sesame oil include oleic acid (43%), linoleic acid (35%), palitic (11%), and stearic (7%). Oleic acid is linoleic’s highest form of fatty acids in sesame seed that is useful (considering the amount of calories needed per day) for the body (Elleuch et al., 2006 ▶). Lignin found in Sesame oil is responsible for many of the physiological and biochemical properties such as antioxidant, anti-mutagenic, and anti-inflammatory. It also improves blood lipid profile and reduces lipid peroxidation in hypercholesterolemic individuals (Rong et al., 2005 ▶). The presence of lignin in sesame prevents DNA oxidative damage in *in* *vivo* systems (Kang et al et al., 1998 ▶). Sesame is a strong antioxidant and long-term treatment of STZ-diabetic animals and has been shown to reduce oxidative stress (Roghani et al., 2011 ▶). Streptozotocin causes testicular dysfunction and degeneration under situations of experimentally induced diabetes in animal models (Shrilatha et al., 2007 ▶). Sesame can decrease STZ’s harmful effects on testis and sperm parameters by reducing reactive oxygen species (ROS). The purpose of the present study was to determine the effects of sesame on spermatogenesis and testicular tissue disorders in STZ-induced diabetic rats.

## Materials and Methods

**Animal treatment**

Thirty adult Wistar male rats (8 weeks old), weighing 200±20 g were purchased from animal facility of Pasteur Institute of Iran, Tehran. Male rats were acclimatized in temperature controlled rooms (25 °C) with constant humidity (40-70%) with 12/h light and 12/h dark cycles for one week. 

All experiments were conducted in accordance with the Institutional Guidelines for the Care and Use of Animals for Experimental Purposes. All rats were fed a standard diet and water. The rats were randomly selected and divided into control (C) (n=10) and diabetic groups that received 65 mg/kg (i.p.) streptozotocin (STZ) (n=20). The rats were then sub-divided into two groups of 10, diabetic control group (DC) and sesame-treated diabetic group (SD). SD group received 100 mg/kg sesame extract (i.p.). C and DC groups just received an equal volume of 1 ml distilled water daily (i.p.). Diabetes was induced by a single intraperitoneal (i.p.) injection of STZ. Sesame injections were continued to the end of the study (for 6 weeks). Forty-two days later (at the end of the treatment period) the rats were anesthetized by diethyl ether and the testes in the control and experimental groups were immediately removed. 

*Induction of experimental type 1 diabetes*

After an overnight fasting, experimental type 1 diabetes was induced by intraperitoneal (i.p.) injection of 65 mg/kg streptozotocin (STZ, Sigma, U.S.A.) in 0.1 M citrate buffer (pH 4.5). Three days after STZ injection, development of diabetes was confirmed by measuring glucose level in fasting blood samples taken from tail vein using Accu-Chek glucometer (Roche, Germany) (Sancheti et al., 2010 ▶). Rats with blood glucose concentrations of 300 mg/dl or higher were considered diabetic and included in the study. Blood glucose levels of the control rats remained normal (<100 mg/dl). (Hosseinzadeh et al., 2002 ▶)

**Preparation of aqueous extracts of sesame seeds**

Sesame seeds were authenticated by a professor from the Department of Biology at Urmia University (Herbarium number: 6071)

One hundred grams of powder samples were added to 1000 ml ethanol 96%, then after 24 h, the solution was filtered. In the second step, ethanol 70% was added to the remained dry materials. After 24 h, the solution was filtered and then evaporated repeatedly to half of the first volume by rotary evaporator in 50 ^o^C and 70 rpm. Concentrated extracts were dried on water bath at 40 ^o^C temperature to prepare the extract for injection. This powder was solved in specific volume of normal saline and later used for the *in vivo* study (Boskabady et al., 2006 ▶).

**Serum sampling**

Blood samples (approximately 4-5 ml blood from each rat) were collected directly from heart in centrifuge tubes without anticoagulants and allowed to clot. The clotted blood was then centrifuged at 3000× g for 10 minutes. Serum was separated and then quickly stored at -80 °C for biochemical analysis.

**Histological analysis**

*Measurement of seminiferous tubule diameter*

Mean diameter of the tubules was measured in 25 random tubules using a coulometer and calculated with the following formula mean = large diameter is the length and small diameter is the breadth of the tubule multiplied magnification.

$\sqrt[{2}]{\mathrm{smalldiameter}\times \mathrm{largediameter}\times \mathrm{magnification}}$

Ten most circular seminiferous tubules were randomly identified in each section of the testis, and their diameters were measured with an ocular micrometer using the 10× lens. The mean seminiferous tubule diameter in micrometers was determined for each testis (Soudmany et al., 2005 ▶).

*Examination of spermatogenesis*

To calculate the spermiogenesis index, it was investigated and confirmed that seminiferous tubules contain sperm compared with seminiferous tubules without any sperm (Shetty et al., 2000 ▶).

*Examination of* *the number of* *Sertoli cells*

According to Abdullahnejad et al. (2009) ▶, 25 tubules in each group at each cutting sections were selected under a microscope and the average number of sertoli cells were calculated.

*Biochemical indices*

The estimation of serum testosterone, LH, and FSH were carried out using the commercial kit purchased from Amersham International. All tests were performed according to the manufacturer’s instructions.

**Testes**

The testes were initially dissected out of a whole via midline abdominal incision and cleared of fats and blotted dry. Their weights were measured on a sensitive digital balance scale with volume measured by water displacement using a 10-ml measuring cylinder. Then the sizes (length and width) were recorded using a sliding gauge (d=0.1). Eventually, they were fixed in freshly prepared 10% normal saline solution as earlier described Abdullahnejad et al. (2009) ▶. Two testes from each rat were measured and the average value obtained for each of the parameters was regarded as one observation.

**Statistical analysis**

Statistical analyses were carried out using SPSS 16 software (SPSS/PC-16, SPSS Inc., Chicago, IL, USA). The data were expressed as mean±SEM. The one-way analysis of variance (ANOVA) followed by Tukey’s post-hoc test were used for analysis of data. The p<0.05 was considered statistically significant.

## Results

**Histological observations**

**Figure. 1 A, B:**
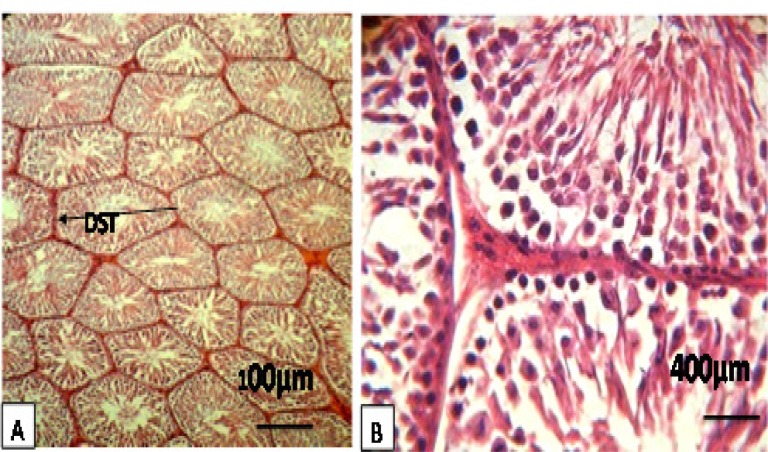
Rat testes after hematoxylin-eosin staining and ×100, ×400. Figures display representative images for a healthy control. It show images of the diameter of seminiferous tubules (DST), thickness of the epithelium, and spermiogenesis index (SPI). This observation indicates normal interstitial tissue, Sertoli cells, and thickness of the epithelium. Their seminiferous tubules showed all of the distinct developmental stages of spermatogenesis, including spermatozoa in the lumen of the tubules

Texture studies showed that in the control group (C), seminiferous tubules were completely healthy in terms of appearance as well as all levels of spermatogenic cells which were observed. The thickness of the basement membrane was also normal (Figure 1, Table 1).

**Figure 2-A, B.**
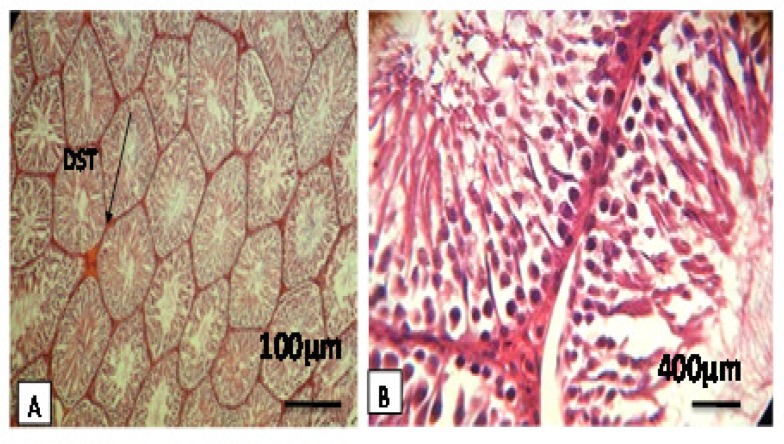
Rat testes after hematoxylin-eosin staining and 100×, 400×. Figures display representative images for a treatment with sesame. They show images of the diameter of seminiferous tubules (DST), thickness of the epithelium, and spermiogenesis index (SPI). This observation indicates an increase in interstitial tissue, thickness of the epithelium, Sertoli cells, and diameter of seminiferous tubules, Moreover, Developmental stages of spermatogenesis can be seen

On the other hand, in the diabetic control (DC) group, it was observed that in seminiferous tubules, the cellular levels have been reduced of spermatocytes and spermatids and the connections between cells have been disappeared. Increasing the thickness of basement membrane was observed and the spaces between seminiferous tubules were quite clear. Moreover, atrophy was observed in leydig cells (Figure 2, Table 1).

**Figure 3A, B.**
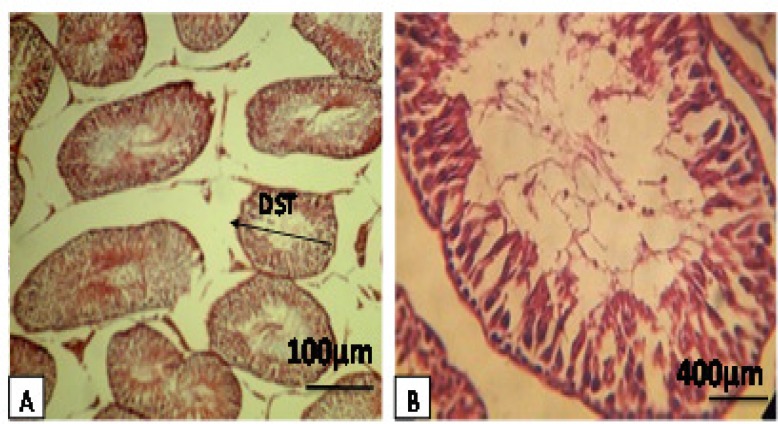
Rat testes after hematoxylin-eosin staining and 100×, 400×. Figures display representative images from a diabetic rat showing the diameter of seminiferous tubules (DST), thickness of the epithelium, and spermiogenesis index (SPI). This observation indicates a loss of interstitial tissue, Sertoli cells, developmental stages of spermatogenesis, and also reduction of sperm in the lumen and diameter of seminiferous tubules

In the diabetic group treated by sesame (SD), it was observed that the appearance of all tubules were normal in a way that all cellular levels (spermatocytes and spermatids) were normally visible. Furthermore, the interstitial space between tubules was quite natural. Leydig cells were also in normal conditions and a decrease in the thickness of the basement membrane was seen (Figure 3, Table 1). In this study, we observed a significant reduction (p<0.001) in testicular weight in the DC group compared with the C and SD groups. Moreover, a significant reduction (p<0.05) in testicular diameter (mm), length (mm), and volume (cm^3^) in DC group compared with the C and SD groups was observed (Table 2). Histological analysis showed that the animals in DC group exhibited a remarkable (p<0.05) reduction in seminiferous tubules diameter, while sesame-treated rats significantly had elevated diabetes-decreased tubular diameter. The data for morphometric analysis are presented in (Table 3).

Moreover, a significant difference (p<0.001) in thickness of the epithelium tissue was observed between DC with the control and SD groups (Table 3). Spermiogenesis index (SPI) was significantly (p<0.05) decreased in DC group in comparison with the C and SD groups (Table 3). 

The mean number of Sertoli cells in the diabetic group shows a significant reduction (p<0.001) compared with the C and SD groups (Table 3).

**Biochemical changes**

The biochemical analysis manifested that the animals in the SD and C groups had a significantly (p<0.001) higher serum level of FSH in comparison with the diabetes-induced group (Table 4). 

The serum level of testosterone was significantly (p<0.05) decreased in the diabetic control rats compared with the non-diabetic rats. Sesame-treated diabetic rats showed a significant increment (p<0.05) in testosterone level compared with the diabetic control rats (Table 4). The serum level of LH was significantly (p<0.001) decreased in the diabetic control rats compared with the non-diabetic rats. Treatment with sesame extract significantly increased the LH level in diabetic rats. No significant effect of sesame extract in non-diabetic rats was observed (Table 4). The serum level of glucose was significantly (p<0.001) increased in the diabetic control rats compared with the non-diabetic rats. Treatment with sesame extract significantly (p<0.001) decreased the glucose level in the diabetic rats (Table 4). 

**Table 1:** Comparison of changes in testicular tissue in different groups. Each group consisted of ten animals. -Absent, + slight, ++moderate, +++ severe

**Group**	**Reduced number of spermatocytes**	**Reduced number of spermatids**	**Atrophic tubules**	**Increased thickness of the basement membrane**
**Control (C)**	-	-	-	-
**Control-diabetes (CD)**	+++	+++	+++	++
**Sesame extract-diabetes (SD)**	-	-	+	+

**Table 2:** The effects of sesame extract on weight, diameter, length and volume of testicular of STZ-induced diabetic rats.

**Group**	**Testis weight (g)**	**Testicular diameter (mm)**	**Testicular length (mm)**	**Testicular volume (cm3)**
**Control (C)**	1.26±0.07	12.58±0.51	23.92±01.04	1.74±0.23
**Control-diabet (CD)**	0.68±0.056٭	8.65±0.69٭	18.85±0.66٭	0.72±0.16٭
**Sesame extract-diabet (SD)**	1.21±0.07	11.69±0.65	23.54±1.01	1.21±0.08

Data are expressed as mean±SEM. Each group consisted of ten animals.

* Significant difference (p<0.05) compared with C and SD group.

**Table 3:** Effects of sesame extract on seminiferous tubules diameter (STD), spermiogenesis index (SPI), thickness of the epithelium, and Sertoli cell in streptozotocin-induced diabetic rats

**Group**	**STD**	**Thickness of the epithelium**	**SPI**	**Sertoli cell**
**Control**	112.19±0.97	45.32±0.7	94.75 ± 0.95	41.5±1.29
**Control-diabet(CD)**	63.5±1.29٭	29.84±0.9 ٭٭	54.00 ±1.82٭	14. ±0.81٭٭
**Sesame extract diabet (SD)**	112.01±1.06	42.98±0.8	93.50 ±1.29	40.75±0.95

Data are expressed as mean±SEM. Each group consisted of ten animals.

٭٭ Significant difference (p<0.001) compared with C and SD group,

٭ Significant difference (p<0.05) compared with group C and SD group.

**Table 4:** Effects of sesame extract on testosterone, LH, and FSH of streptozotocin-induced diabetic rats

**Group**	**Testestrone ** **ng/ml**	**FSH** **IU/L**	**LH** **IU/L**	**Glucose** **mg/dl**
**Control**	3.63 ± 0.257	0.14±0.192	0.23±0.009	96.25±1.5
**Control-diabet (CD)**	2.80 ± .175٭	0.06±0.199٭٭	0.13±0.005٭٭	365.75±1.7٭٭
**Sesame extract-diabet (SD)**	3.59 ± 0.242	0.13±0.19	0.22±0.008	98.75±0.95

Data are expressed as mean±SEM. Each group consisted of ten animals.

٭ Significant at (p<0.05)

٭٭ Significant at (p<0.001).

## Discussion

Several reports showed that sexual behavior and reproductive tract functions are markedly affected by diabetes mellitus which can lead to reduced fertility (Palmeira et al., 2001). The present study showed that diabetes increased blood glucose and created extensive histological changes in rats and treatment with sesame extract improved testis tissue damage by protection of seminiferous tubules, spermatogenic cells, and sertoli cells. Diabetes causes the reduction of spermatogenic cells and decreases the tubules diameter by cell apoptosis and seminiferous tubules atrophy (Guneli et al., 2008 ▶). These changes are indicative of morphologic disorders in spermatogenesis (Cai et al., 1997 ▶; Cameron et al., 1985 ▶). 

In this research, atrophy and reduction of seminiferous tubules diameter and spermatogenic cells were seen in the diabetic group. Diabetes increases the thickness of basal lamina in somniferous tubules which accompanies reduction of sperm production and total size of somniferous tubules (Rohrbach et al., 1982 ▶). That is compatible with last researches about sesame effects which showed increasing of spermiogenesis tubule’s diameter (Predes et al., 2007). Moreover, reduction in Sertoli cells causes reduction in sperm number. Sertoli cells have an important role in spermatogenesis in providing physical and nutrition protection and necessary hormone signals for successful spermatogenesis (Okamura et al., 2004 ▶), therefore, when Sertoli cells reduce, the number of germinal cells decreases intensively (Richburg et al., 2000 ▶). 

In the present research, it was observed that diabetes caused reduction of Sertoli and germinal cells in the treatment group with sesame extract that can directly reveal positive effects of sesame oil on testis tissue. It has been reported that size of the testis is related to Sertoli cells number and sperm production so that the size of testis is reflecting the germinal cell numbers (Slegtenhorst-Eegdeman et al., 1998 ▶). Sesame prevents tubules diameter and Sertoli cells number and spermiogenesis rate reduction and causes reduction of diameter, length, and weight of testis. Sesame does not increase the interstitial tissue but its effect was impressive in the diabetic group. Diabetes effect on tissue function is due to inadequate insulin production and subsequent reduction of effect of that hormone deteriorates Sertoli and Leydig cells function. Moreover, reduction of insulin levels weakens the spermogenesis process by reduction of FSH rate (Kiyanifard et al., 2010 ▶).

In recent studies, a direct relationship between testosterone and gonadotropins has been reported. FSH and LH stimulate the production of androgens. Low blood levels of these hormones can play an important role in the reduction of testosterone production by the testes tissue. Moreover, LH is needed for Leydig cells activity (Kiyanifard et al., 2010 ▶).

On the other hand, hyperglycemia causes oxidative stress because of the increased level of reducing sugars. These reducing sugars can easily react with lipids and proteins, thus the production of oxygen reactive species (ROS) increases that gradually leads to the development of diabetic complications (Moody et al.,2008 ▶).

In STZ-induced diabetes, due to the effect of ROS on Leydig cell function, testosterone level decreases which is responsible for the alterations found in the seminiferous epithelium of diabetic animals (Shahreari et al., 2010 ▶)

Reduced level of testosterone hormone in diabetic rats and changes in structure and function of Sertoli cells can affect normal function of Leydig cells (Sarkar et al., 2000 ▶). Another probable mechanism in dysfunctioning of Leydig cells can be increasing of free radicals and oxidative stress that can prevent androgens production by Leydig cells (Aitkem et al., 1989 ▶). Germinal cells health and their ability for mitotic divisions in seminiferous tubules relates to testosterone secretion by Leydig cells. Disorder in testosterone biosynthesis in Leydig cells has harmful effects on male fertility (Yang et al., 2007). Reduction of testosterone production is responsible for histological changes in testis (Bairy et al., 2009 ▶). In the treatment group with sesame, significant increase of testosterone level was evident compared with the diabetic group. 

The reports state that decrease of testosterone rate and gonadal abnormalities cause inadequate sperm production. 

 The study showed that sesame extract by reducing blood glucose level and reducing oxidative stress (due to having high antioxidant content) has positive effects on morphology and hormone alterations in STZ-induced diabetic rat.
